# Supplementary material for: The Smiles of Older People through Recreational Activities: Relationship between Smiles and Joy
Source: Int J Environ Res Public Health. 2021 Feb 9;18(4):1600. doi: 10.3390/ijerph18041600 (PMC7914444; doi:10.3390/ijerph18041600)
Supplement: Supplementary file 1 [file ijerph-18-01600-s001.pdf]

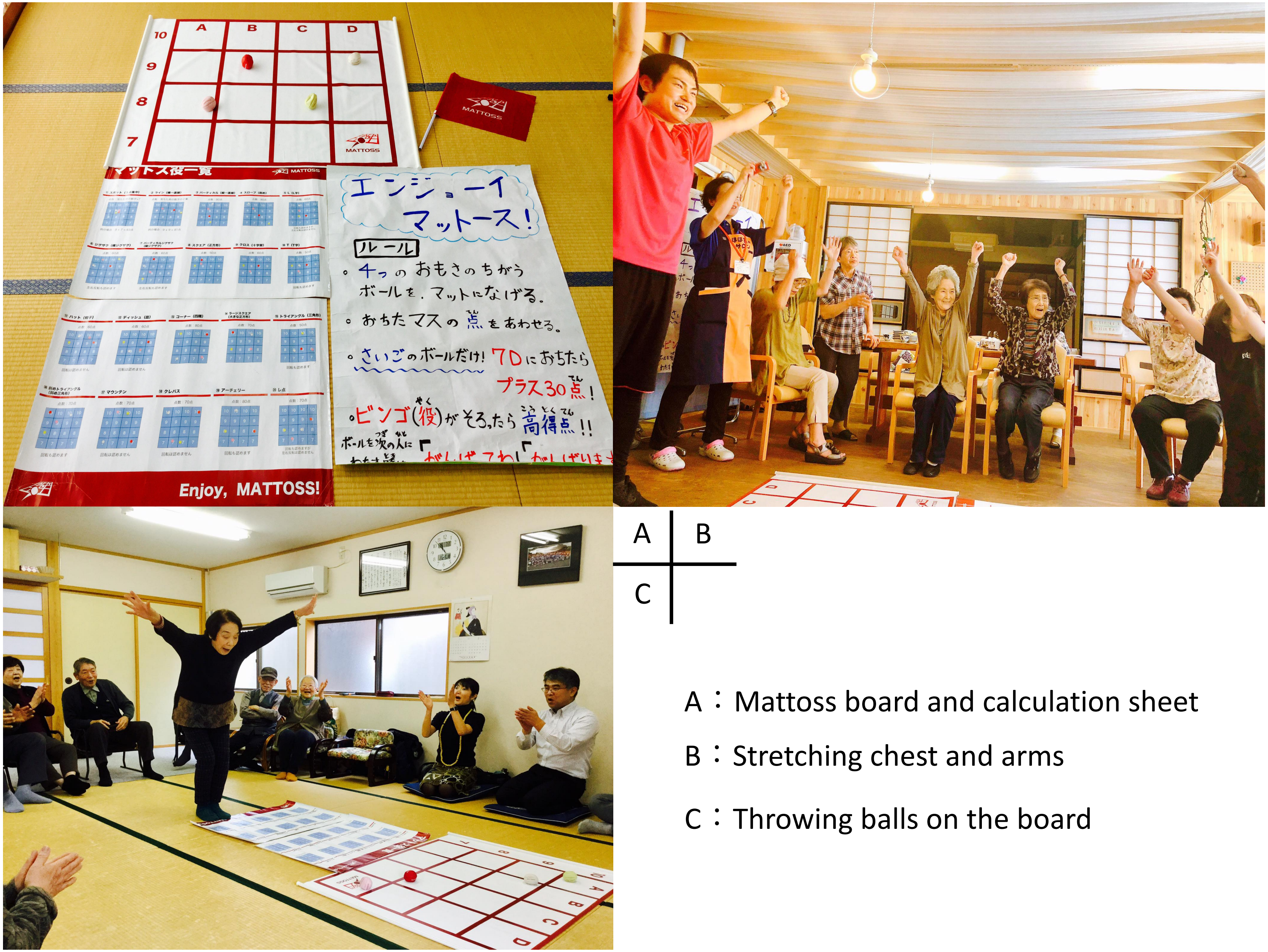

A：Mattoss board and calculation sheet

B：Stretching chest and arms

C：Throwing balls on the board

Figure 1. Pictorial illustrations of Mattoss.

Copyright © 2020 community nurse company All Rights Reserved.
